# The Relationship Between Bronchial Patency and Parameters of ECG Supraventricular Component in Children With Bronchial Asthma

**DOI:** 10.3389/fped.2020.00576

**Published:** 2020-09-16

**Authors:** Alina V. Gordina, Ksenia A. Egoshina, Tatyana I. Eliseeva, Nadezhda G. Vinogradova, Dmitry Yu. Ovsyannikov, Elena V. Tush, Andrey V. Prakhov, Mojisola I. Daniel-Abu, Olga V. Khaletskaya, Nailya I. Kubysheva

**Affiliations:** ^1^Privolzhsky Research Medical University, Nizhny Novgorod, Russia; ^2^City Clinical Hospital No. 38, Nizhny Novgorod, Russia; ^3^Department of Pediatrics, Peoples' Friendship University of Russia (RUDN University), Moscow, Russia; ^4^Research Laboratory “Clinical Linguistics”, Kazan Federal University, Kazan, Russia

**Keywords:** bronchial patency, ECG, supraventricular component, bronchial asthma, children

## Abstract

**Background:** Uncontrolled asthma (BA) can be complicated by cardiac conduction disturbances and arrhythmias. It is typical mainly for adult asthmatics patients. In asthmatics children the effect of bronchoconstriction on cardiac conduction, including the supraventricular component of the ECG, is currently under discussion. The objective of the research is to analyze ECG parameters of the atrial complex and atrioventricular conduction and to assess their relationship with spirometric indicators in children with BA.

**Methods:** Hundred three patients with BA from the age of 6–17 years were examined. The spirometric parameters were evaluated, including the Tiffeneau index (TI): FEV1/FVC (%), according to the level of which the patient groups were distinguished. Group 1 (G1): with TI more than 85%, (*n* = 15); Group 2 (G2): with TI from 85 to 75%, (*n* = 40); Group 3 (G3): with TI <75%, (*n* = 48). The ECG parameters that characterize supraventricular conduction, including the PQ interval (sec) and the sPQ segment (sec), were analyzed. We had calculated relative PQ (rPQ) by the formula rPQ=PQ/PQmed, where PQ is the patient's PQ, PQmed are the median PQ values of healthy children of age selected.

**Results:** The duration of the PQ in groups G1 and G2 was 0.13 (0.11; 0.14) s; and 0.13 (0.12; 0.14) s, respectively, which is statistically significantly less than in patients of groups G3–0.14 (0.13; 0.15] s, *p* = 0.01. The duration of the sPQ segment in children of groups G1 and G2 was also generally shorter than in patients of groups G3, and amounted, respectively, to 0.05 (0.04; 0.06) s, 0.04 (0.04; 0.05) s, and 0.06 (0.04; 0.07) s, *p* = 0.02. The rPQ increased progressively as TI decreased and amounted in G1 to 92.9 (85.7; 106.3) %, in G2 100.0 (92.9; 103.0) %, and in G3 104 (100.0; 107.7) %, *p* = 0.009. A statistically significant negative correlation between IT and PQ–*r* = −0.23, *p* = 0.02; with sPQ–r = −0.20, *p* = 0.045; and with rPQ–*r* = −0.25, *p* = 0.01 was revealed.

**Conclusion:** A decrease in TI in asthmatics children is associated with a prolongation of the PQ. This may indicate a slowdown in supraventricular conduction in patients with uncontrolled asthma and, thus, be considered as a risk for the formation of subsequent supraventricular arrhythmias.

## Introduction

Bronchial asthma (BA) is a chronic inflammatory disease of the respiratory tract, the leading clinical manifestations of which are reversible bronchoconstriction and bronchial hyperreactivity (1–3). The goal of BA therapy at this stage is achieving control over symptoms and risk factors of exacerbation of the disease (4–6). However, the course of BA can be complicated by various comorbidities, which have a negative influence on the achievement of control (7–12).

That said, insufficient control of BA, in turn, can cause the formation of various pathological conditions. For example, there are studies showing the risk of cardiac arrhythmias and conduction disorders in patients with uncontrolled BA due to functional changes or pathological remodeling of the myocardium (13–16). Atrial remodeling, which is the pathomorphological basis of serious supraventricular cardiac arrhythmias, has a more rapid progression with poor BA control and is formed as a result of excessive stretching of the atrial wall, as well as other adverse factors (17).

The connection between BA and supraventricular arrhythmias, including atrial fibrillation (AF), was noted in studies by Warnier et al. (18), Goudis et al. (19), and Cepelis et al. (20). Available data indicate that in the adult population, cardiac arrhythmias are significantly more common in patients with BA than in those without it (13, 18, 21, 22). The results of a Norwegian population study HUNT study, demonstrate that the risks of supraventricular arrhythmias and AF are increased in patients with an uncontrolled BA (20).

Despite the fact that AF is diagnosed primarily in adult patients with BA, other supraventricular arrhythmias are described in patients with childhood BA (18, 23–25). At the same time, there are a number of studies demonstrating the development of atrial remodeling and changes in the electrophysiological properties of the atria of children with BA (23, 26). Probably, one of the reasons for the significant impairment of the electrophysiological properties of the atria may be impairments of bronchial patency. Thus, the prerequisites for the development of severe supraventricular arrhythmias in patients with BA may be formed in childhood. Therefore, identifying markers of the risk of supraventricular arrhythmias in children with BA will help predict their development and prevent cardiac complications of asthma, particularly in its uncontrolled form, in both children and adults.

Consequently, it should be noted that in the study of Çiftel et al. it was found that patients with BA are characterized by disorders of electrophysiological properties of the right atrium, which consist of an increase in the intra-atrial and interatrial conduction time. This, according to the authors, can be considered as a predictor of supraventricular arrhythmias in patients with BA, the risk of which increases as the severity and duration of uncontrolled asthma increases (26).

Electrocardiography (ECG) is the universal screening method for assessing the state of the atrial myocardium and the conducting system of the heart. According to German et al. ECG analysis can make a significant input to the assessment of the risk of formation of supraventricular rhythm and conduction disorders. Therefore, the analysis of the atrial component of the ECG, and atrioventricular conduction in patients with BA is an important component of the management of these patients, especially in pediatric practice (27). Consequently, the study of the characteristics of the ECG and its supraventricular component in children with BA is relevant.

Considering the available data on the reduced rate of atrial conduction in patients with an insufficient level of asthma control, it is important also to study the relationship between ECG parameters that characterize the atrial complex and atrioventricular conduction, and spirometric parameters that reflect the severity of bronchial obstruction, an important component in the formation of clinical manifestations of asthma (28, 29). However, to date, the evaluation of the ECG atrial component and atrioventricular conduction in patients with childhood BA remains insufficiently researched. Studies dedicated to the evaluation of the supraventricular component of ECG in relation to the state of bronchial patency in children with BA, which would allow us to identify predictors of myocardial remodeling and supraventricular rhythm disorders in children and to predict the development of these complications of BA, were not found in the literature.

The purpose of this study, therefore, is to analyze the electrocardiographic parameters of the atrial complex and atrioventricular conduction and evaluate their relationship with spirometric indicators in children with bronchial asthma.

## Materials and Methods

### Formation of a Cohort of Patients

The study was carried out in accordance with the Helsinki Declaration adopted in June 1964 (Helsinki, Finland), and revised in October 2000 (Edinburgh, Scotland). The study was approved by the Ethics Committee of the Privolzhsky Research Medical University, Protocol No. 13 of 10.10.2016. Informed consent was obtained from patients aged 15–17 years, and from the parents of patients under the age of 15 years, in accordance with Federal law No. 323 of 21.11.2011 “On the basics of health protection of citizens in the Russian Federation.”

Hundred three children aged 6–17 years were examined, of which, boys made up 67.6% (71/103), and girls−32.4% (32/103), These patients were treated for bronchial asthma in the Nizhny Novgorod City Children's Clinical Hospital No. 1.

The inclusion criteria were a diagnosis of BA, made in accordance with current international and national consensus documents, and the presence of a sinus rhythm based on the results of an ECG analysis (3, 30). The exclusion criteria were: saturation below 98%, the presence of acute infectious diseases and fever (31), diabetes, autoimmune disorders, primary immunodeficiency and cancer, oral glucocorticoids.

BA treatment was carried out in accordance with existing consensus documents, taking modern therapeutic strategies into account (1, 3, 6, 12).

### Objective Measurements

In addition to general clinical and allergy examinations, all patients underwent spirometric and electrocardiographic tests, as well as the measurement of the main anthropometric indicators. Saturation was evaluated using the pulse oximetry method (medical pulse oximeter “Armed” YX302, China). Spirometric tests were performed using the MasterScreen Pneumo spirometer (Jaeger, Germany), in accordance with existing international recommendations (32). Also assessed were the forced vital capacity (FVC), forced expiratory volume per second (FEV_1_), and maximal expiratory flow at 25% of the flow-volume curve (MEF_25_); data were recorded in absolute values and in relative units, i.e., percentages of reference values (% predicted—hereafter % pred), subject to gender, age and anthropometric indices of the child. Also, the Tiffeneau index (FEV_1_/FVC)^*^100% was evaluated (3, 32–34).

The Tiffeneau Index (TI) was used as a marker for bronchial patency. A TI value >85% (3, 33), indicated that bronchial patency was unimpaired or slightly impaired, with TI values <85% but more than 75%, moderate bronchial obstruction was considered, and with TI values <75%, patients were considered to have serious impairment of bronchial patency (3, 33). ECG recording was performed on a multichannel Cardiofax S electrocardiograph (NihonKonden, Japan) at a paper speed of 50 mm/s. The atrial complex of the electrocardiogram and atrioventricular conduction were analyzed based on the height of the *P*–wave (mm), the total duration of *P*-wave (sec), the duration of PQ interval (sec), the duration of the segment sPQ (sec) in the II standard lead. The value of sPQ (sec) was determined as the difference between PQ (sec) and duration of *P*-wave (sec). Also evaluated were the heart rate (HR, beats per minute), and the duration of the RR interval (sec). The relative value of interval PQ (rPQ) was also calculated, using the formula: rPQ=PQ/PQmed, where PQ is the duration of PQ interval of the patient, and PQmed is the median duration of PQ interval, typical for children of this age and gender (32). Also, the fraction of the PQ interval (sec) in the total duration of the cardiac cycle (R–R, sec) was calculated–PQ/RR × 100%.

### Statistical Analysis

Since this was a pilot study, the sample size was not calculated. Statistical analysis was done using the Statgraphics Centurion V. 16.1.17 software package. The data is presented in the form of Me [A1; A2], where Me is the median, [A1; A2] are the lower and upper bounds of the 95% confidence interval.

For quantitative features, standardized skewness and standardized kurtosis were calculated to determine the normality of the sample. Quantitative data for which the calculated values of standardized asymmetry and standardized kurtosis were outside the range of −2 to +2 were characterized as different from normal values. Samples of indicators such as age (years), FVC (liters), FVC% (%pred), heart rate (BPM), ampP (mm), *P* (sec), rPQ (%), sPQ (sec), rHR (%), RR (sec) were normal.

Differences between two independent samples with normal distribution were determined using the Student *t*-test and between three or more independent samples using the *F*-test (ANOVA variance analysis). The differences between two independent samples different from normal ones were calculated using the non-parametric Wilcoxon–Mann–Whitney test (W criterion, comparing the medians of two samples); between three or more independent samples-using the Kruskal–Wallis test (KWT, comparing the medians of several samples). The relationship between independent samples was evaluated using the Spearman correlation coefficient *r*. The level of statistical significance was taken as *p* < 0.05.

## Research Results

### Clinical Characteristics of Patients

The study included children and adolescents from 6 to 17 years of age, with a median age of 11.0 (6.0; 17.0) years. Boys and girls were comparable in age and anthropometric indicators, including height, body weight, and BMI, Table 1.

**Table 1:** Clinical and functional characteristics of the examined BA patients.

**Parameters**	**Boys**	**Girls**	**Statistics**
Number of patients	71	32	
Age, years	11.0 (10.0; 12.0)	11.0 (9.0; 14.0)	*t* = 0.003; *p* = 1.0; *W* = 1138.5; *p* = 0.99
Height, cm	150.0 (141.0; 163.5)	151.0 (143.0;160.0)	*W* = 998.5; *p* = 0.33
Weight, kg	43.5 (35.9; 54.3)	41.5 (29.5; 52.0)	*W* = 974.0; *p* = 0.25
BMI, kg/m^2^	18.5 (17.0; 20.0)	18.5 (15.9; 20.1)	*W* = 998.5; *p* = 0.33
FVC, l	3.24 (2.89; 3.90)	2.94 (2.57; 3.76)	*t* = 2.12; *p* = 0.04; *W* = 893.5; *p* = 0.08
FVC, %pred	113.2 (108.1; 117.0)	113.3 (104.7;126.0)	*t* = −0.33; *p* = 0.74; *W* = 1185.0; *p* = 0.73
FEV_1_, l	2.25 (1.96; 2.82)	2.23 (1.62; 2.83)	*W* = 961.5; *p* = 0.21
FEV_1_, %pred	101.1 (97.5; 104.6)	100.9 (93.0; 111.4)	*W* = 1155.0; *p* = 0.90
Tiffeneau index, %	73.2 (70.7; 77.1)	76.1 (72.0; 81.6)	*W* = 904.0; *p* = 0.07
MEF_25_, l/s	0.86 (0.67; 1.02)	0.82 (0.70; 1.25)	*W* = 1131.0; *p* = 0.83
MEF_25_, %pred	48.3 (43.5; 55.9)	51.6 (41.0; 74.9)	*W* = 1212.0; *p* = 0.42
HR, BPM	75.0 (72.0; 77.3)	80.0 (74.0; 88.0)	*t* = −2.12; *p* = 0.04; *W* = 1438.5; *p* = 0.03
rHR, %	93.2 (90.1; 95.8)	97.7 (92.5; 102.8)	*t* = −2.23; *p* = 0.03; *W* = 1447.5; *p* = 0.03
RR, sec	0.80 (0.78; 0.83)	0.75 (0.68; 0.81)	*t*= 4.74; *p* = 0.03; *W* = 833.5; *p* = 0.03
P, sec	0.08 (0.08; 0.09)	0.08 (0.06; 0.09)	*t* = 0.83; *p* = 0.41; *W* = 1005.0; *p* = 0.34
amp P, mm	1.5 (1.0; 1.5)	1.5 (1.0; 2.0)	*t* = −1.49; *p* = 0.14; *W* = 1314.0; *p* = 0.18
PQ, sec	0.13 (0.13; 0.14)	0.13 (0.12; 0.14)	*W* = 935.0; *p* = 0.14
rPQ, %	100.0 (100.0; 107.1)	92.9 (92.3; 100.0)	*t* = 1.35; *p* = 0.18; *W* = 892.5; *p* = 0.08
sPQ, sec	0.05 (0.04; 0.06)	0.05 (0.04; 0.06)	*t* = 0.22; *p* = 0.82; *W* = 1142.5; *p* = 0.97

*BMI, kg/m2–body mass index; FVC, l–forced vital capacity; FVC, %pred–percentage of predicted FVC; FEV_1_, l–forced expiratory volume in first second; FEV_1_, %pred–percentage of predicted FEV_1_; MEF_25_, l/s–maximal expiratory flow rate at 25% of flow-volume loop; MEF_25_, %pred–percent of predicted MEF_25_; HR, BPM–heart rate; P, sec—duration of P wave; amp P, mm—amplitude of P wave; PQ, sec–duration of PQ interval; rPQ = PQ/PQ med × 100%; sPQ, sec–duration of the PQ segment; rHR = HR/ HR med × 100%; RR, sec–duration of RR interval*.

Spirometry parameters measured in absolute terms and in relative units (% pred) in this sample had no statistically significant gender differences, with the exception of FVC (liters), which was higher for boys than for girls (*p* = 0.04). Indicators characterizing the ECG atrial complex and atrioventricular conduction, including the height (mm) and duration (sec) of the *P*-wave, the duration of the PQ interval (sec), and the sPQ segment (sec) in boys and girls in the study sample were comparable. The median heart rate for girls was higher than for boys, *p* = 0.04. Accordingly, the rHR (%) in the girls' group was higher than in the boys' group (*p* = 0.03). For the same reason, the duration of RR (sec) in girls was less than in boys, *p* = 0.03. Given, however, that most of the studied parameters of both the ECG and spirometry had no gender differences in the available sample, further analysis of indicators was carried out in the total sample of patients without regard to their gender.

We evaluated the relationship of the main parameters characterizing the ECG atrial component, with the age of the patients with BA who were examined, Table 2. It was found that the duration of the intervals PQ (sec) and RR (sec) have a statistically significant relationship with the age of the child, *p* = 0.008 and *p* = 0.0002, respectively. This suggests that considering these parameters as a single sample in a common cohort of children of different ages would be incorrect. Because of this, we have introduced additional relative values to qualify the PQ (sec) and RR (sec) intervals, –*rPQ*, and *rRR*, respectively. These parameters were normalized to the median values after accounting for the age and height of patients (32). Relative parameters are equations: *rPQ*=*PQ/PQmed*, where PQ is the patient's PQ values, PQmed is the median *PQ*-values typical of children of this age; and *rRR*=*RR/RRmed*, where RR is the patient's RR values, RRmed is the median RR values typical of children of this age.

**Table 2:** Relationship between atrial conduction parameters and age (years) of patients, *n* = 103.

**ECG parameter**	***R*, *p***
P, sec	*R* = 0.07; *p* = 0.50
PQ, sec	*R* = 0.26; *p* = 0.0078
sPQ, sec	*R* = 0.14; *p* = 0.16
ampP, mm	*R* = 0.01; *p* = 0.92
RR, sec	*R* = 0.35; *p* = 0.03

*P, sec–duration of P wave; PQ, sec–duration of PQ interval; sPQ, sec–length of PQ segment; amp P, mm–amplitude of P wave; RR, sec–duration of RR interval; R, correlation coefficient; p, level of statistical significance*.

### Indicators of ECG Atrial Component and Supraventricular Conduction in Children With Different Degrees Severity of Bronchoconstriction

**Figure 1:**
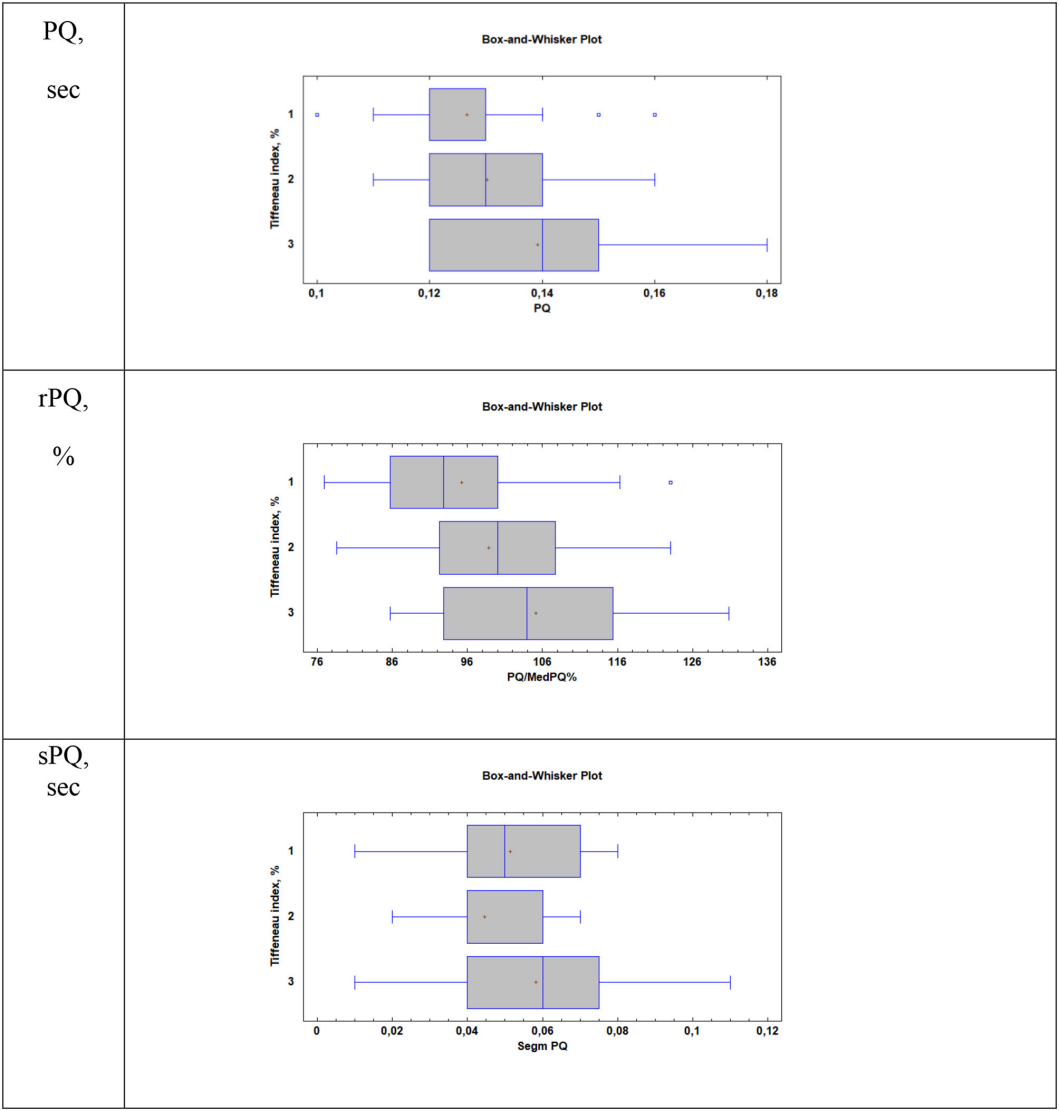
Supraventricular conduction parameters in patients with different values of the Tiffeneau index (R. Tiffeneau) in children with BA. Group 1, Patients with TI >85%; Group 2, Patients with 75% < TI <85%; Group 3, Patients with TI <75%; PQ, sec–duration of PQ interval; rPQ = PQ/PQ med × 100%; sPQ, sec–duration of PQ segment.

When considering atrial conduction parameters, it was found that children who had the most pronounced manifestations of bronchoconstriction (Group 3, Tiffeneau index <75%) had a statistically significant lengthening of the PQ interval (sec), *p* = 0.01; an increase in the rPQ coefficient (%), *p* = 0.009; and an extension of the sPQ segment (sec), *p* = 0.02, compared with patients in Group 1 (Tiffeneau index more than 85%) and Group 2 (Tiffeneau index from 75 to 85%), Table 3, Figure 1. At the same time, no intergroup differences in the values of *P* (sec), RR (sec), rRR (%), and ampP (mm) were established.

**Table 3:** ECG parameters in children with different degrees of severity of bronchoconstriction.

**ECG parameters**	**Group 1**	**Group 2**	**Group 3**	**Statistics**
Number of patients	15	40	48	
*P*, sec	0.08 (0.06; 0.08)	0.09 (0.08; 0.10)	0.08 (0.07; 0.08)	*F* = 1.91; *p* = 0.15; KWT = 4.38; *p* = 0.11
PQ, sec	0.13 (0.11; 0.14)	0.13 (0.12; 0.14)	0.14 (0.13; 0.15)	*F* = 5.66; *p* = 0.005; KWT = 9.07; *p* = 0.01
rPQ, %	92.9 (85.7; 106.3)	100.0 (92.9; 103.0)	104.0 (100.0; 107.7)	*F* = 5.87; *p* = 0.004; KWT = 9.32; *p* = 0.009
sPQ, sec	0.05 (0.04; 0.06)	0.04 (0.04; 0.05)	0.06 (0.04; 0.07)	*F* = 5.10; *p* = 0.008; KWT=8.05; *p*=0.02
RR, sec	0.76 (0.69; 0.87)	0.77 (0.72; 0.81)	0.81 (0.75; 0.88)	*F* = 2.73; *p* = 0.07; KWT = 3.85; *p* = 0.15
HR, BPM	79.0 (69.0; 87.1)	78.0 (74.0; 83.7)	74.5 (68.0; 80.0)	*F* = 1.97; *p* = 0.14; KWT = 3.85; *p* = 0.15
rHR, %	95.8 (87.5; 98.6)	94.7 (92.1; 102.7)	92.5 (83.8; 99.2)	*F* = 1.31; *p* = 0.28; KWT = 2.32; *p* = 0.31
ampP, mm	1.5 (1.0; 2.0)	1.5 (1.0; 2.0)	1.3 (1.0; 1.8)	*F* = 0.61; *p* = 0.55; KWT = 1.01; *p* = 0.60

*Group 1, Patients with TI >85%; Group 2, Patients with 75% < TI <85%; Group 3, Patients with TI <75%. The data is presented in the form of Me [A1; A2], where Me is the median, [A1; A2] is the lower and upper limits of the 95% confidence interval. P, sec–duration of P wave; PQ, sec–duration of PQ interval; rPQ = PQ/PQ med × 100%; sPQ, sec–length of PQ segment; RR, sec–duration of RR interval; HR, BPM–heart rate in beats per minute; amp P, mm–amplitude of P wave*.

When evaluating the relationship between ECG and spirometric parameters, we established a statistically significant positive correlation between the duration of the *P*-wave (sec) and heart rate (BPM) with such absolute indicators of external respiration as FVC (l), FEV_1_ (l), Table 4. A similar relationship was also established between the duration of the PQ interval (sec) and the value of FVC (l). This seems to reflect the influence of age and anthropometric parameters of children and adolescents on some ECG indicators, as well as on the absolute values of spirometric parameters.

**Table 4:** Relationship between spirometry parameters and ECG atrial component in children with BA.

	**FVC, l**	**FEV_**1**_, l**	**MEF_**25**_, l/s**	**Tiffeneau index, %**	**FEV_**1**_, %pred**	**MEF**_**25**_, **%pred**
*P*, sec	*r* = 0.20; *p* = 0.046	*r* = 0.20; *p* = 0.04	*r* = 0.17; *p* = 0.09	*r* = 0.02; *p* = 0.82	*r* = 0.09; *p* = 0.39	*r* = 0.08; *p* = 0.44
PQ, sec	*r* = 0.24; *p* = 0.02	*r* = 0.13; *p* = 0.19	*r* = −0.04; *p* = 0.70	*r* = −0.23; *p* = 0.02	*r* = −0.21; *p* = 0.03	*r* = −0.16; *p* = 0.10
sPQ, sec	*r* = 0.007; *p* = 0.94	*r* = −0.08; *p* = 0.41	*r* = −0.18; *p* = 0.07	*r* = −0.20; *p* = 0.045	*r* = −0.23; *p* = 0.02	*r* = −0.19; *p* = 0.05
HR, BPM	*r* = −0.32; *p* = 0.001	*r* = −0.24; *p* = 0.01	*r* = −0.12; *p* = 0.23	*r* = 0.13; *p* = 0.19	*r* = 0.09; *p* = 0.37	*r* = 0.02; *p* = 0.83
ampP, mm	*r* = −0.11; *p* = 0.28	*r* = −0.11; *p* = 0.25	*r* = −0.06; *p* = 0.55	*r* = 0.02; *p* = 0.87	*r* = −0.18; *p* = 0.07	*r* = −0.06; *p* = 0.58
rPQ, %	*r* = −0.02; *p* = 0.83	*r* = −0.12 *p* = 0.23	*r* = −0.22; *p* = 0.03	*r* = −0.25; *p* = 0.01	*r* = −0.20; *p* = 0.04	*r* = −0.25; *p* = 0.01
rHR, %	*r* = 0.04; *p* = 0.66	*r* = 0.09; *p* = 0.36	*r* = 0.11; *p* = 0.26	*r* = 0.09; *p* = 0.35	*r* = 0.04; *p* = 0.66	*r* = 0.09; *p* = 0.35

The data is presented as R (p), where R is the correlation coefficient, and p is the level of statistical significance.

*P, sec–duration of P wave; PQ, sec–duration of PQ interval; sPQ, sec–duration of PQ segment; HR, BPM–heart rate, beats/min; ampP, mm–amplitude of P wave; rPQ^.^= PQ/PQ med × 100%; rHR, HR/HRmed × 100%; FVC, l–forced vital capacity; FEV_1_, l–forced expiratory volume in the first second; MEF_25_, l/s–maximal expiratory flow rate at 25% of flow-volume loop; FVC, % pred–percentage of predicted FVC; FEV_1_, %pred–percentage of predicted FEV_1_; MEF_25_, % pred–percentage of predicted MEF_25_*.

In this study, a statistically significant negative correlation between the duration of the PQ interval (sec) and sPQ (sec), and relative spirometrics parameters, that characterize bronchial patency, including TI (%) and FEV_1_ (%pred) was established. A statistically significant negative correlation was also established between rPQ (%) and spirometry indicators such as MEF_25_ (%pred), TI (%), FEV_1_ (%pred), and MEF_25_ (l/s). The results obtained suggest that the worsening of bronchial obstruction in children with BA may be accompanied (characterized) by an extension of the PQ interval, both in absolute and relative units.

## Discussion

This study is aimed at studying the effect of bronchial obstruction on supraventricular conduction in children with BA. Using the method of spirometry and analysis of ECG parameters, it was found that in children with BA, as the Tiffeneau index decreases, there is a statistically significant lengthening of the PQ interval and the sPQ segment, as well as an increase in the rPQ coefficient, which apparently, indicates a slowing of the conduction of impulses from the atria to the ventricles as bronchoconstriction increases. It should be noted that the study did not include children who had a saturation below 98% during the survey period, which completely excluded the presence of hypoxia in children.

Parameters of the supraventricular component of the ECG (PQ, sPQ segment, rPQ) were statistically significantly higher in patients who had severe manifestations of bronchial obstruction (Tiffeneau index lower than 75%) compared with children who had no bronchial obstruction or were from mild to moderate (Tiffeneau index higher than 75%), all *p* < 0.05. At the same time, in our study, no differences in the amplitude, shape, and duration of the P wave in patients with different degrees of severity of bronchoconstriction were observed; the median values of *P*-wave duration in all three groups with different values of the Tiffeneau index were close to 0.08 sec, all *p* > 0.05. This is consistent with the data of Ghandi et al. (23), in whose study, on comparing patients with BA with healthy children, it was found that the maximum and minimum duration of the *P*-wave were identical in subjects of both groups, although the dispersion of the *P*-wave was higher in patients with asthma. However, this is not entirely consistent with the data of Yucel et al. whose study revealed a tendency to lengthen the maximum duration of the P wave when analyzed in 12 ECG leads in children with asthma compared to healthy children (35). Thus, the evaluation of the *P*-wave in patients with asthma still requires more detail, with the expansion of the patient group and stratification of patients by age, gender, and severity of bronchoconstriction.

Increased duration of supraventricular conduction (lengthened PQ interval), as well as delayed atrioventricular conduction (lengthened sPQ segment) detected in patients with BA who have spirometric signs of obvious bronchoconstriction, require close attention. The data obtained during the ECG analysis are consistent with the results obtained in a study by Ciftel et al. to assess the impact of the severity of bronchial obstruction on the function of the conduction system of the heart in children with BA. Using the tissue Doppler echocardiography method, the authors found that patients with BA have a statistically significant increase in interatrial and right atrial conduction times, compared to healthy children (26). Similar patterns of increased right atrial conduction time were observed in patients with COPD, which may be proof that prolonged supraventricular conduction time associated with bronchoconstriction is a universal phenomenon (36). On the other hand, as shown by Daubert et al. prolonged intra-atrial and inter-atrial conduction time are also typical for patients with atrial arrhythmias, including atrial fibrillation (AF) (37).

Thus, it cannot be ruled out that increased supraventricular conduction time in patients with significant bronchoconstriction can be considered as a potential predictor of the formation of subsequent supraventricular arrhythmias, including AF. This is consistent with the results of a study by Shibata et al. showing that respiratory dysfunction in patients with BA is an independent risk factor for AF (38). The mechanism of formation of AF in patients with obstructive pulmonary diseases, however, remains largely unclear. This hinders the development of reliable programs for the prevention of these serious arrhythmias in patients with BA (38).

Distension and dilation of the atria are among the important pathophysiological mechanisms for the formation of supraventricular cardiac arrhythmias. As demonstrated by Schotten et al. (39), this leads to the uneven supraventricular spread of impulses from the sinus node, as well as the formation of re-entrant circuits inside the atrium. Currently, new data have been made available about the oneness of the cardiopulmonary system, including the presence of cardiomyocytes in pulmonary vein tissue, as described in a review by Folmsbee and Gottardi (40). The relationship between certain BA phenotypes and the electromechanical properties of the heart has also been demonstrated. This may, in the future, serve as a starting point for treatment aimed at achieving BA control, being targeted toward corresponding cardiorespiratory mechanisms (40).

It is also known that the cardiac conduction system is affected by hypoxia, causing pronounced electrophysiological changes in the myocardium (impaired repolarization, decreased electrical activity) (41). Myocardial dystrophy develops, accompanied by reduced cardiac output and impaired acid-base balance, which is a trigger for arrhythmias (42). However, patients who had a saturation below 98% were not included in this study.

Electrocardiographic changes that accompany an increase in airway obstruction may also be due to autonomic mechanisms. Respiratory homeostasis is known to be controlled by the sympathetic and parasympathetic nervous systems. In BA, there is an increase in the tone of the parasympathetic nervous system, meanwhile the abnormal activity of the parasympathetic nervous system can be closely associated with the pathogenesis of asthma and is reflected in heart rate variability (43, 44). Parasympathetic influences slow down the heart rate and prolong PQ. However, our data indicate only PQ lengthening without associated bradycardia. The vagal effect is probably not the only mechanism of delayed conduction of supraventricular impulses.

Taking into account the pilot character of the current study, it is useful to estimate the sample size necessary for validating of the found peculiarities. The sample size *N* can be crudely estimated using the known formula *N* = 2 · (*Z*_α_+*Z*_β_)^2^/(*d*/*SD*)^2^, where *Z*_α_ and *Z*_β_ are the normal distribution values at the levels of type I and II error probabilities of α/2 and β, respectively; *d/SD* is the required (clinically-significant) ratio between the group mean value difference (*d*) and their standard deviations (*SD*). Using the typical values of α = 0.05 for 95% confidence level and β = 0.80 for criterion power level, *Z*_α_ and *Z*_β_ values are 1.96 and 0.84, respectively. Considering Group 1 (with normal FEV values) and Groups II+III (with increased FEV values) as two groups with normal and elongated *rPQ* values, the *d/SD* value in our study is of about 0.82. In order to obtain the more significant results with *d/SD*=0.2, the same design study should be conducted in the cohort of 392 patients, and in the cohort of 1,568 patients for *d/SD* = 0.1 (45).

## Conclusion

In this study, it was found that the duration of the PQ interval and sPQ segment in children with BA who had pronounced spirometric manifestations of bronchial obstruction, were higher than in patients who had no impaired bronchial patency. The disclosed elongation of the PQ interval and sPQ segment in children with pronounced spirometric markers of bronchial obstruction cannot be ignored, since it is known that in adult patients with AD, the progression of the disease can be accompanied by the formation of supraventricular cardiac arrhythmias, including serious and fatal ones. This study should be repeated with an increased sample size and supplemented by long-term follow-up of patients, including monitoring of spirometric bronchial patency and measurement of ECG parameters.

## Data Availability Statement

All datasets generated for this study are included in the article/supplementary material.

## Ethics Statement

The studies involving human participants were reviewed and approved by the study was approved by the Ethics Committee of the Privolzhsky Research Medical University, Protocol No. 13 of 10.10.2016. Informed consent was obtained from patients aged 15–17 years, and from the parents of patients under the age of 15 years, in accordance with Federal law No. 323 of 21.11.2011. On the basics of health protection of citizens in the Russian Federation. Written informed consent to participate in this study was provided by the participants' legal guardian/next of kin.

## Author Contributions

TE conceived of the study, including design. AG, KAE, AP, and ET were responsible for data collection and data interpretation. NK and MD-A performed the statistical analysis. All authors read and approved the final manuscript.

## Conflict of Interest

The authors declare that the research was conducted in the absence of any commercial or financial relationships that could be construed as a potential conflict of interest.
